# Sulfur Metabolism of the Gut Microbiome and Colorectal Cancer: The Threat to the Younger Generation

**DOI:** 10.3390/nu15081966

**Published:** 2023-04-19

**Authors:** Ji-Yeon Moon, Bong-Hyeon Kye, Seung-Hyun Ko, Ri Na Yoo

**Affiliations:** 1 Division of Colorectal Surgery, Department of Surgery, St. Vincent’s Hospital, College of Medicine, The Catholic University of Korea, Suwon 442-723, Republic of Korea; 2 Division of Endocrinology and Metabolism, Department of Internal Medicine, St. Vincent’s Hospital, The Catholic University of Korea, Suwon 442-723, Republic of Korea

**Keywords:** colorectal neoplasm, exposome, Western diet

## Abstract

Colorectal cancer diagnosed in individuals under 50 years old is called early-onset colorectal cancer (EOCRC), and its incidence has been rising worldwide. Simultaneously occurring with increasing obesity, this worrisome trend is partly explained by the strong influence of dietary elements, particularly fatty, meaty, and sugary food. An animal-based diet, the so-called Western diet, causes a shift in dominant microbiota and their metabolic activity, which may disrupt the homeostasis of hydrogen sulfide concentration. Bacterial sulfur metabolism is recognized as a critical mechanism of EOCRC pathogenesis. This review evaluates the pathophysiology of how a diet-associated shift in gut microbiota, so-called the microbial sulfur diet, provokes injuries and inflammation to the colonic mucosa and contributes to the development of CRC.

## 1. Introduction

Colorectal cancer (CRC) is the third most diagnosed cancer and the second leading cause of cancer mortality globally [1]. CRC incidence parallels socioeconomic advancement or human developmental index (HDI) over time [2]. Previous studies have indicated that the higher levels of HDI are likely related to changes in the prevalence of lifestyle-related risk factors, including increased consumption of red and processed meats and refined carbohydrates, obesity, physical inactivity, alcohol consumption, and smoking [3,4]. To reduce CRC incidence and mortality, many countries have adopted colonoscopy screening programs for early detection and prevention by re-moving precancerous polyps during colonoscopy [5,6,7]. The overall CRC incidence has declined in countries such as the USA, Israel, and Japan, where early detection programs have been established since the 1990s [5,8].

However, a global assessment of contemporary trends in CRC incidence indicated that CRC incidence rates have significantly increased among young adults younger than 50 over the past two decades [9]. The CRC diagnosed in individuals under 50 years old is called early-onset colorectal cancer (EOCRC). The reasons for such a steep escalation of the development of EOCRC are ambiguous. Nevertheless, in addition to the inherited traits, risk factors contributing to the development of EOCRC are similar, but not limited, to those related to the older population, including westernized diet, obesity, and sedentary lifestyle [2,7,9]. Compared to late-onset CRC (LOCRC), the clinical presentation of EOCRC is often more advanced, and the prognosis is less favorable [10]. Therefore, it is essential to understand the underlying mechanisms and to attribute risk factors and unique characteristics of EOCRC for early diagnosis and appropriate intervention.

Emerging evidence suggests that diet is a significant factor associated with gut microbial activity related to a rise in EOCRC [11]. The high-fat, high-sugar diet, known as the Western diet, alters the genetic composition and metabolic activity of the human gut microbiome, particularly involving sulfur metabolism [11]. This review explores the temporal changes in the epidemiology of CRC incidence correlating to global food consumption. It examines the effect of dietary components on intrinsic changes in gut microbial composition and their metabolic activity. Furthermore, this review evaluates the pathophysiology of how a diet-associated shift in gut microbiota provokes injuries and inflammation to colonic mucosa and contributes to CRC development, particularly early in life.

## 2. The Worrisome Trend in CRC Incidence among Young Adults

**Figure 1:**
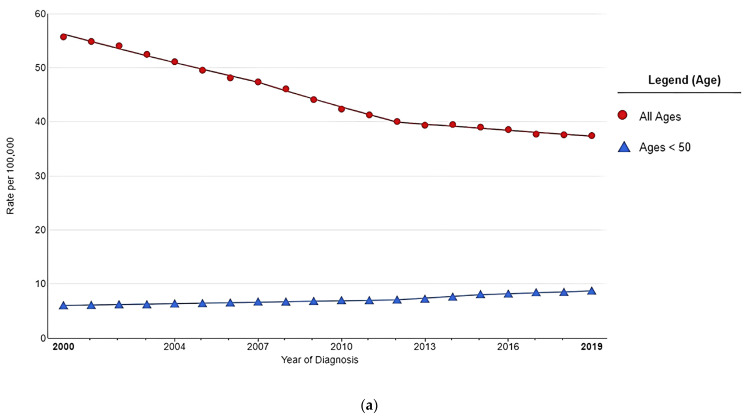

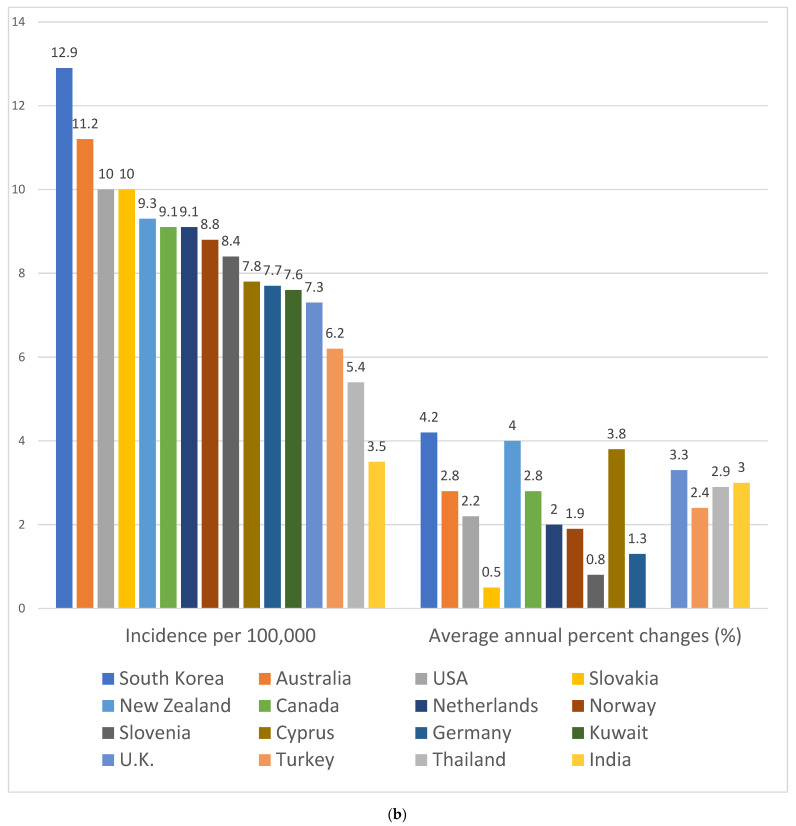
(**a**). A recent trend in SEER Age-adjusted colorectal cancer incidence rate from 2000 to 2019. This figure was created by https://seer.cancer.gov/statistics-network/explorer (accessed on 15 February 2023). (**b**) Increase in early-onset colorectal cancer incidence rate. This graph is created based on the summary of the published articles by Siegel, R.L. et al. [9].

According to the U.S. cancer registry data from 2013 to 2017, 54% of CRC cases are diagnosed at an age greater than 65 years and 34% between 50 and 64 years, with the median age at 67 years [12]. After adopting an age-targeted screening program with colonoscopy and rising public awareness of CRC risk factors, the incidence rates have rapidly declined, by 3.3% annually in the U.S., among elderly individuals [12,13,14]. In contrast, the incidence of CRC in younger adults aged <50 years demonstrated an inclination of 4.3% annual percent change during 2012–2015 and 2.2% during 2015–2019 (Figure 1a) [15]. Moreover, a global analysis of CRC incidence demonstrates that EOCRC is increasing in men and women in 19 countries on five continents [9]. Although most CRC diagnoses remain in older adults over 50, CRC constitutes a significant disease burden in younger adults <50 worldwide (Figure 1b) [1,9,16].

**Figure 2:**
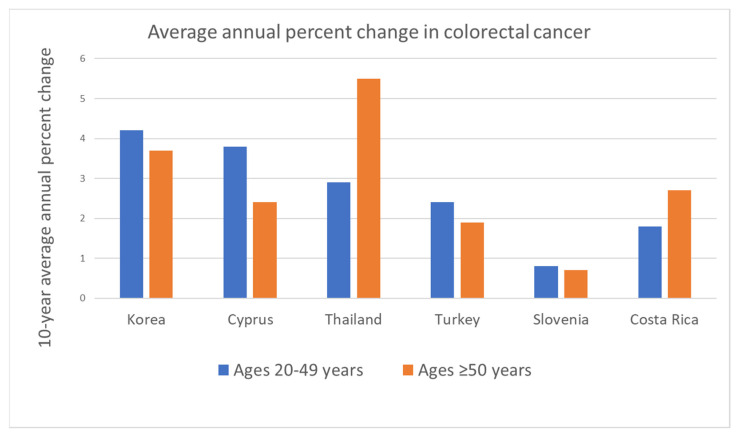
The countries demonstrate increasing colorectal cancer incidence rates in both younger and older populations. This graph is created based on the summary of the published articles by Sung, H. et al. [1] and Siegel, R.L. [12].

The upward trend in the incidence of EOCRC started in the 1990s across the successive birth cohort of people born in and after the 1960s [9,17]. Based on CRC incidence trends between 1975 and 2010 in the USA, the annual percent change-based predicted incidence rates of colon cancer in 2030 will increase by 27.7% for the age of 35 to 49 and 90% for the age of 20 to 34 [18]. The incidence rate of rectosigmoid and rectal cancer is predicted to be even higher—than that of colon cancer—46% for those aged 35 to 49 and 124.2% for those aged 20 to 34 [18]. Such a steep inclination in EOCRC incidence is also observed in countries that have experienced or are currently experiencing rapid industrialization, such as Korea, Cyprus, Thailand, Taiwan, etc. [1,3,9,19,20]. Interestingly, as shown in Figure 2, the EOCRC increment in these countries coincides with the increase in LOCRC during 2008–2012. The simultaneous change in CRC incidence reflects a rapid change in lifestyle and diet that has affected older adults and the younger population over the past several decades [9].

Unmeasured exposures occurring in early life, or exposures frequently experienced by younger generations in countries with very high HDI, may increase the risk of EOCRC and can be represented as the birth cohort effect on EOCRC incidence [17]. Additionally, the rise of CRC for overall ages in countries with a swift shift of development status suggests that exposure to certain risk factors during a timeframe of life might have triggered the expression of CRC after a latent period [17]. The greatest fear is that predisposition to carcinogens early in life, from the prenatal to adolescence, might exert mutagenic damage during the developmental period, possibly resulting in a delayed effect on EOCRC incidence [21]. The cancer trend may portray a heavy disease burden in the future if urgent intervention is not initiated for the younger generation.

## 3. Difference in Clinical and Molecular Features between LOCRC and EOCRC

The clinical features of EOCRC differ from those of elderly patients in terms of stage, tumor location, and histology at the initial diagnosis. Diagnosis of CRC in young adults is usually made when symptoms, predominantly hematochezia, are present [22,23,24,25]. 80.5% of patients complained of various symptoms, such as abdominal pain, bowel obstruction, anemia, and bowel habit change [26]. Despite symptom complaints, a low level of suspicion by a primary physician and reluctance to seek medical care by young patients may delay the diagnosis by a mean of 6.2 months in younger adults < 50 years compared to older adults ≥ 50 years [24,27,28,29]. At diagnosis, patients < 50 years old appear to present advanced stages with nodal or systemic metastasis and are more likely to develop metachronous or distant metastasis in the course of the disease compared to patients ≥ 50 years old [10,23,24,25,26,27,28]. While the anatomical location of LOCRC is dispersed across the colon and rectum at similar frequencies, EOCRC shows a skewed distribution—most commonly in the rectum, followed by the left-sided colon, then the right-sided colon, at 42%, 31%, and 27%, respectively [30,31,32]. Furthermore, compared to LOCRC, the histopathological characteristics of EOCRC are more likely to present aggressive features of high-grade, poorly differentiated tumors with signet-ring or mucin-producing cells, often accompanied by perineural and lymphovascular invasion [10,32,33].

The molecular features of EOCRC are highly heterogeneous, comprising hereditary CRC syndromes resulting from various germline mutations and nonhereditary or sporadic cases without strong familial clustering [17,33,34]. Hereditary EOCRC comprises approximately 5% to 16% of EOCRC cases, affecting individuals early in life, mainly at the age of 20 to 30 years [35,36,37]. The pathogenesis of hereditary EOCRC is associated with germline genetic mutations causing genetic instability, cell proliferation, or dysregulated microenvironment [38]. Unlike hereditary EOCRC, the molecular characteristics of sporadic EOCRC are perplexing and may exhibit unique features distinguishable from those of hereditary EOCRC [39,40]. Although hereditary cancer occurs at a higher rate in a younger population than in an older population, sporadic CRC accounts for 80% of EOCRC cases and is the most common form [17]. Table 1 demonstrates the clinical and molecular features of sporadic EOCRC different from LOCRC. Compared to the LOCRC cases, the oncogenic molecular features for sporadic EOCRC frequently bear microsatellite stability and lack DNA repair mechanism abnormalities which differ in gene expression and molecular pathogenesis [30,36,39,41]. Microsatellite-stable EOCRC exhibits a significant difference in gene expression and molecular pathogenesis from LOCRC [39]. MSS EOCRC shows overexpression of the catenin beta 1 (CTNNB1) gene and association with upregulation of Wnt/beta-catenin, mitogen-activated protein kinase, growth factor signaling, and the tumor necrosis factor receptor 1 pathways, potentially influencing metastasis and chemo-radiosensitivity [42]. Other molecular aberration distinctive to EOCRC includes epigenetic alterations, most commonly long interspersed nuclear elements (LINE-1) hypomethylation, associated with increased chromosomal instability [43]. Diverse molecular alteration profiles exist in EOCRC and remain under active investigation. Nevertheless, given that the most commonly occurring form of EOCRC cases is sporadic, the implication of genetic mutation in pathogenesis is inexplicable. Interaction between environmental factors and genetic predisposition would be essential for tumor expression.

## 4. Lifestyle-Related and Environmental Risk Factors Associated with EOCRC

**Figure 3:**
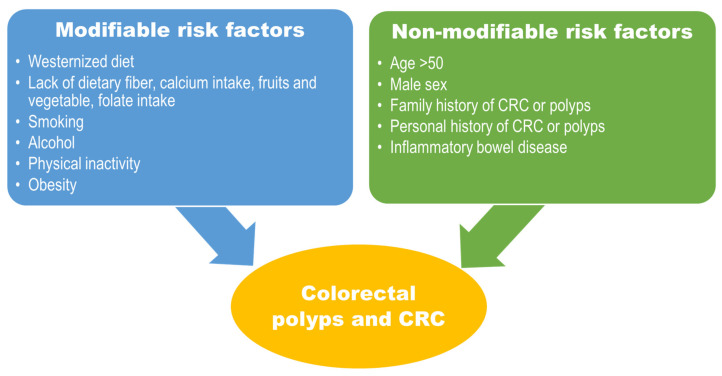
Risk factors can be categorized as modifiable and nonmodifiable.

Risk factors for CRC have been well characterized based on many population-based cohort and case-control studies, primarily categorized into modifiable and nonmodifiable, as shown in Figure 3. Although the risk factors are reported mainly from the older population, data on the younger population indicate that, as in elderly individuals, risk factors related to the Western diet and sedentary lifestyle pose a significant increase in developing CRC [44]. Westernized diets characterized by high fat, high sugar intake with processed or red meat and physical inactivity symbolize human development and industrialization, which are tightly linked to obesity and obesity-related chronic disease or malignancy [2,3,45,46]. This phenomenon is well reflected by the trend of CRC incidence in Asia, particularly China, Japan, South Korea, Singapore, and Taiwan, with a two- to fourfold increase [1,47]. Furthermore, there are notable disparities in EOCRC incidence by geography and ethnicity in the United States [48]. High incidences are observed in regions like the southern states, Mississippi Delta and Appalachia [17]. Regarding racial disparity, the rise of EOCRC incidence is notable in Hispanic/Latino men and Whites, although CRC incidence has previously been high in African-American men [48]. Such disparities partly reflect poverty, unemployment, and poor access to the healthcare system among the younger population [48]. Nevertheless, easy access to poor-quality diets could contribute to the rising incidence of EOCRC.

**Figure 4:**
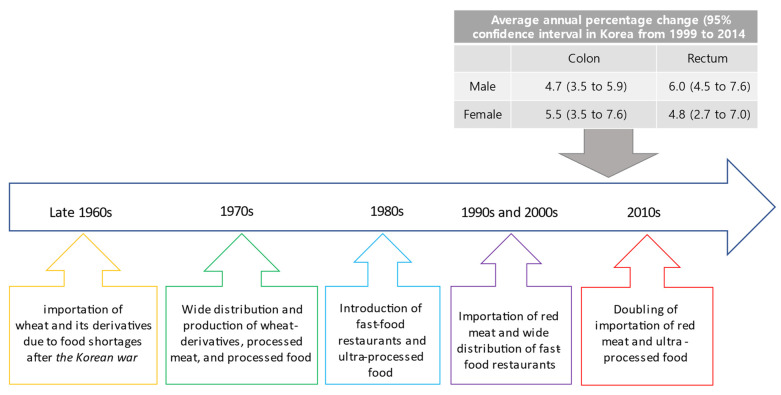
Dietary transition and the incidence of early-onset colorectal cancer.

Following spectacular economic growth, a fast dietary transition to increased consumption of highly refined wheat and its derivatives, processed or red meat, and ultra-processed food took place for the past several decades in South Korea, as shown in Figure 4 [49,50]. It is not surprising that the prevalence of obesity increased from 6.8% to 10.0% in Korean children and adolescents aged 6 to 18 from 1998 to 2013 [51]. At the same time, type 2 diabetes among children 18 years or younger increased from 153.5 per 100,000 in 2006 to 205.0 per 100,000, a relative increase of 33.6% [51]. Furthermore, evaluating physical activity, screen time, and sleep duration, a cross-sectional study using national data indicated that only 1.6% of adolescents met the recommendation from the Canadian 24-h Movement Guidelines for Children and Youth, which consists of at least 60 min of moderate-to-vigorous physical activity, no more than 2 h of screen time, and 8–11 h of sleep duration over a typical 24-h day [52]. Worse yet, another study evaluating the 6-year prevalence trend adhering to the recommendation demonstrated that fewer than 1% of adolescents met all three recommendations consistently [53]. It is evident that early-life exposures to known risk factors, such as consuming ultra-processed food and insufficient physical activity, have prevailed among children and adolescents in Korea. Given that a long latency period for normal colonic mucosa to transform into cancer is necessary, profound physiologic and metabolic derangement starting early in life partly explains the increase in the incidence of sporadic EOCRC [54,55,56].

## 5. Diet as Exposomes Associated with EOCRC

**Figure 5:**
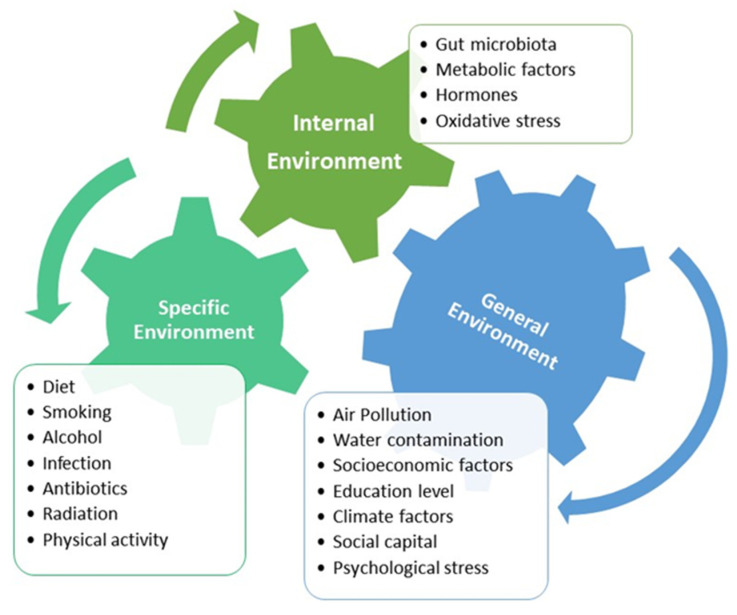
Three components of exposomes for colorectal cancer.

The advancement of genetic research and molecular epidemiology shows that environmental life-course exposures to risk factors play a fundamental role in disease expression [57]. In 2005, Wild suggested the concept of “exposome”, the individual’s environmental exposure from the prenatal period onward, as it matches the individual’s genome [58]. The exposome consists of three overlapping domains: the general external environment, the specific external environment, and the internal environment, as shown in Figure 5 [39,59]. It is difficult to place a particular exposure in one domain or another and to measure how much a particular exposure can cause disease in an individual when considering lifespan and different developmental periods [59]. However, epidemiologic studies have attempted to establish risk factors at a population level, and molecular pathological epidemiology of epigenetics reveals certain aberrant epigenetic signatures in identifying disease characteristics, particularly malignant neoplasms [57]. Epigenetic alterations, such as LINE-1 hypomethylation or CpG island methylator phenotype, are often associated with EOCRC [32,57]. Although specific exposomal data related to EOCRC are limited, an abundance of epidemiologic data and molecular research on CRC indicates that dietary habit, use of antibiotics, chemical exposures, smoking, and alcohol as exposomes have complex interactions with endogenous gut microbiota and host factors, stimulating inflammation, cell proliferation, and genetic mutation [40].

Dietary components profoundly affect the composition of the gut microbiota and may shift dominant bacterial colonies within the gut microbiome, influencing host metabolism and immunity [60]. Previous in vitro and murine studies showed that a diet high in animal protein increases Bacteroides species, Alistipes species, and Bilophila species which are bile-tolerant microorganisms while decreasing bacterial species that metabolize dietary plant polysaccharides, such as Lactobacillus, Roseburia, Eubacterium rectale, and Bacillus bifidus [61]. Additionally, a diet high in fat appeared to increase Firmicutes and Mollicutes but reduce Bacteriodetes, increasing metabolites, such as lipopolysaccharides (LPS), trimethylamine-N-Oxide (TMAO), and reduction of short-chain fatty acids (SCFA) [62,63]. These metabolites are often the metabolic byproducts of microorganisms from the “Western diet”, high in fat and sugar and associated with chronic low-grade inflammation and metabolic disturbance, causing insulin resistance, obesity, and diabetes [61,63].

Similar findings have been shown in human studies. A study using biopsy samples from colonic mucosa and fecal samples in African Americans who had two-week food exchanges from a Western diet to a high-fiber diet indicated a remarkable increase in saccharolytic fermentation and butyrogenesis while suppressing secondary bile acid synthesis, which correlates to a protective effect on the colonic mucosa and lowers CRC risk [64]. A dietary intervention study using human fecal samples demonstrated that bacterial colonization could be shifted swiftly depending on the type of diet, either animal-based or plant-based, and alteration in microbiota might elicit transcriptional responses of gene abundance from the dominant microbiome [11]. As ultra-processed food has been widely distributed and easily accessed, detrimental effects from dominating microbiota adapted to the Western diet can be exerted during the developmental period. This exposomal element of diet may explain the surge of sporadic EOCRC, in which a relatively long process of carcinogenesis reflects decades of exposure to poor dietary elements causing a pathogenic shift in the microbiome and deleterious metabolism [65].

Intestinal dysbiosis can initiate chronic inflammatory conditions of the colonic mucosa, produce carcinogenic metabolites, and even cause direct DNA damage [60,66,67]. Previous studies using metagenomic data on the microbiome associated with a colorectal polyp or CRC showed that certain strains of bacteria are found at a higher frequency in patients with precursor adenoma or cancer, such as Fusobacterium nucleatum, enterotoxigenic Bacteroides fragilis, and polyketide synthase gene complex (pks+) *Escherichia coli* [68,69,70]. These bacteria may cause direct DNA damage, modulate-cadherin/β-catenin, and promote a tumor-permissive microenvironment by recruiting myeloid-derived suppressor cells and inhibiting the antitumor immunity of NK or T cells [71,72,73]. These pathogenic mechanisms of bacteria are often associated with sporadic CRC arising from the adenoma-carcinoma sequence [74].

## 6. Sulfur Metabolism of Gut Microbiota and Its Association with CRC Development

Hydrogen sulfide (H_2_S) is widely accepted as a critical signaling molecule in humans, identified as a gasotransmitter with various chemical properties, reaction mechanisms, and the ability to alter proteins and participate in many metal redox processes [75]. Endogenous H_2_S is predominantly produced by gut microbiota from metabolizing inorganic sulfur (sulfate and sulfite) from preservatives in processed food and organic sulfur compounds, mostly cysteine or taurine from red meat [76,77]. Sulfate-reducing bacteria, such as Bilophila, Desulfovibrio, Desulfomicrobium, and Fusobacterium, can colonize the gut in the human intestinal tract and generate endogenous H_2_S from metabolizing inorganic or organic sulfur compounds [66,78]. Several microbial enzymes, including cystathionine β-synthase (CBS), cystathionine γ-layse (CSE), and 3-mercaptopyruvate sulfurtransferase (3-MST), are responsible for the production of endogenous H_2_S from catabolizing cysteine and homocysteine [78].

As H_2_S is produced from microbial metabolic reactions, luminal H_2_S permeates easily through the biofilms that cover the colonocyte and epithelial cell membrane due to its high permeability [79]. Entering the colonocytes, H_2_S is catabolized through intracellular oxidative metabolism in the mitochondria and cytoplasm [80]. Composed of several mitochondrial enzymes in the colonocyte, including sulfide quinone oxidoreductase (SQR), ethylmalonic encephalopathy protein 1 (ETHE1), and thiosulfate thiotransferase (TST), the sulfide oxidation unit oxidizes H2S and produces persulfides, a highly reactive molecule binds to proteins [81]. This physiological post-translational modification of proteins (S-sulfuration) is known to regulate and affect the processes of cell survival and death, cell differentiation, cell proliferation and hypertrophy, cellular metabolism, mitochondrial bioenergetics and biogenesis, vasorelaxation, inflammation, oxidative stress [82]. It has been shown that the S-sulfuration regulates the DNA damage repair system by activating the RAS–RAF–MEK–ERK cascade by sulfhydrated MEK1, influencing tumor growth [83,84]. Additionally, the persulfidation to the NF-κB induces metastasis-promoting gene expression and activates NF-κB/IL-1β signaling, which may result in cancer progression and metastasis via VEGF activation [85].

**Figure 6:**
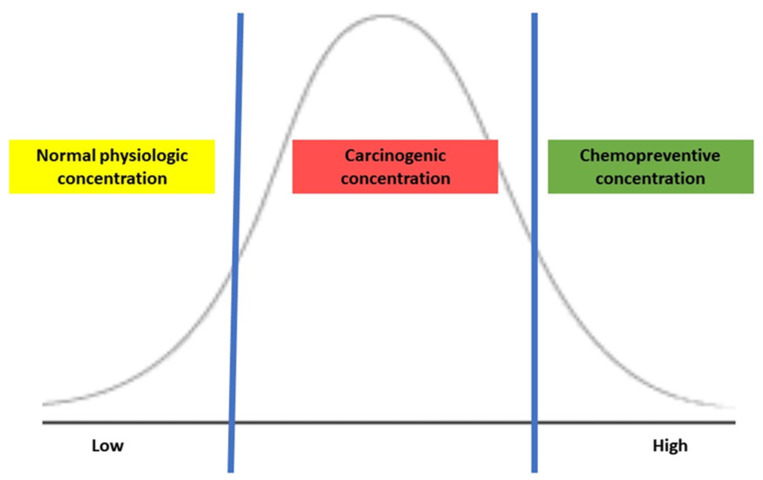
The action of H_2_S is based on its concentration.

The biological effects of H_2_S depend on its concentration in the colonic lumen, and the luminal concentration is mainly determined by endogenous production by bacterial metabolism, which influences H_2_S-mediated tumorigenesis. Several in vitro studies of treating CRC cell lines with exogenous H_2_S reveal bell-shaped concentration responses in cancer, representing the dual effects of H_2_S [75]. When CRC cells were exposed to a slow-release H_2_S donor at a low concentration (0.2–0.3 micromole), mitochondrial function and glycolysis for energy production enhanced cancer cell proliferation by activating H_2_S-generating enzymes within cancer cells but were typically not present in colonic epithelial cells [86,87]. Additionally, the expression of H_2_S-producing enzymes was higher in CRC tissue than in normal surrounding tissues, possibly maintaining its optimal concentration for tumor growth and proliferation [85]. Conversely, treating CRC cells with a high concentration (1 millimole) of an H_2_S donor in the form of isothiocyanate, a molecular derivative from a cruciferous plant, induced the apoptosis of CRC cells [88]. As shown in Figure 6, the exogenous H_2_S demonstrates a concentration-dependent effect: maintenance of normal physiology at low, carcinogenic once reaching an upper threshold, then possibly chemopreventive at a high. Thus, maintaining an appropriate concentration of H_2_S may be critical to balance the cell cycle and regulating apoptosis and tumorigenesis.

The upregulation of H_2_S and sulfidogenic bacteria positively correlates with a diet high in fat and protein [66,89]. A high concentration of sulfidogenic bacteria in stool is associated with the risk of distal CRC [90]. Moreover, comparing the flatus samples from patients with CRC to those from healthy individuals, the concentration of the sulfur compounds was significantly higher in the patients with CRC [91]. In vitro study using colon cancer-derived epithelial cell lines demonstrated selective upregulation in the ability of H_2_S-producing enzymes, which increased H_2_S concentration compared to the nonmalignant colonic mucosa cells [85]. In mice with loss of the H_2_S-producing enzyme function, the blood flow to the tumor was decreased, inhibiting tumor growth and angiogenesis [85]. The level of CBS in human samples is low in the healthy colonic mucosa but gradually increases as the epithelial cells are transformed into polyps, hyperplastic polyps, tubular adenoma, and adenocarcinoma [92]. The CBS protein levels in human colon cancer specimens closely correlated to the disease severity and tumor stage, and more advanced tumors expressed higher levels of CBS with higher expression of vascular endothelial growth factor (VEGF) [93,94]. Furthermore, it has been shown that expression of H_2_S-detoxifying enzymes, e.g., TST, located in colonocytes in the lumen is markedly reduced in advanced colon cancer [95]. A meta-analysis flowchart by identifying differentially expressed genes among normal colonic mucosa, primary tumor sites, and metastatic samples in the liver and lung demonstrated that the expression of mitochondrial oxidation enzymes, including SQR, ETHE1, and TST, decreased during the evolving process from the normal epithelium to the primary tumor and metastatic lesions [96]. These findings suggest that dysregulated expression and activity of sulfide-detoxifying or -producing enzymes may contribute to disruption in the homeostasis of the sulfur-containing compound. Consequently, increased endogenous H_2_S concentration may play a role as a tumor growth factor, inducing tumor growth and proliferation and promoting angiogenesis and vasorelaxation.

Interestingly, H_2_S can have dual effects, harmful or beneficial, depending on its source and concentration. In a previous in vitro study evaluating the underlying mechanism of H_2_S action causing carcinogenesis, sulfide at concentrations similar to those in the human colon (e.g., millimole) induced direct genomic DNA damage in mammalian cells [97]. Furthermore, H_2_S can cause mucosal damage by breaking disulfide bonds in the mucus layer. Consequently, luminal bacteria and their metabolites can penetrate the epithelial lining, induce apoptosis of epithelial cells, and activate the inflammatory cascade [76,98]. This evidence is consistent with the finding that a Westernized diet increases CRC, particularly in the distal location where sulfur-metabolizing bacteria are found at a higher concentration than in the proximal colon [90]. Intriguingly, some studies have demonstrated that H_2_S has a protective and reparative effect on the colonic epithelium. Endogenous H_2_S at a low concentration (e.g., micromole) can act as a vasorelaxant, reduce endoplasmic reticulum stress, and prevent apoptosis [99]. Additionally, exogenous H_2_S exists in garlic, onions, and cruciferous vegetables, such as cabbage, cauliflower, kale, and broccoli, which are known to be beneficial to colonocytes and enterocytes, acting as an energy source for microbial metabolism. Inorganic plant-derived H_2_S helps colonocyte respiration and stimulates mitochondria to detoxify and recover from epithelial injury [75]. Thus, oral consumption of exogenous H_2_S stabilizes gut microbiota biofilm integrity and prevents the formation of the pathogenic shift in colonies, eventually inhibiting inflammation and tumorigenesis [100]. However, the specific mechanism of H_2_S action connected to the interaction between dietary sources and gut microbiota needs further investigation.

This unique biological property of H_2_S provides new approaches to CRC treatment, targeting H_2_S modulation by delivering exogenous H_2_S in high doses or inhibiting endogenous H_2_S expression [75]. Researchers have developed exogenous H_2_S compounds that can release in a site-specific and time-dependent manner. Various biocompatible polymers of H_2_S have been developed as donors, demonstrating the ability to specifically target the lesions, respond to the pathological microenvironment, and monitor changes in the microenvironment after the delivery [101]. H_2_S-releasing non-steroidal anti-inflammatory drugs (H_2_S-NSAIDS) have been proposed as anticancer drugs [102]. After covalently attaching H_2_S to NSAIDS, the researchers tested the growth properties of different human cell lines from six different tissues. They found that H_2_S-NSAIDS inhibited the growth of all cancer cell lines studied, with potencies of 28- to >3000-fold greater than traditional NSAIDS [102]. HS-NSAIDs inhibited cell proliferation, induced apoptosis, and caused G(0)/G(1) cell cycle block [102]. Additionally, inhibition of endogenous H2S production mainly focuses on targeting enzymes related to endogenous H_2_S synthesis [103]. Several small molecule inhibitor models have been designed and synthesized to inhibit CBS, CSE, and 3-MST, mainly inducing anti-proliferative activity [75]. Aminooxyacetic acid (AOAA) is a well-known CBS inhibitor that reacts with vitamin B6, transforming vitamin B6 into a biologically inactive form [103]. Because CBS requires a biologically active cofactor derived from vitamin B6, pyridoxal-5′-phosphate (PLP), CBS is inhibited in the presence of AOAA [103]. Another attractive approach to reducing endogenous H_2_S concentration is the development of endogenous H_2_S scavengers [103]. For example, hydroxocobalamin has been investigated as a potential scavenger for H_2_S overdose [104]. At all concentrations, hydroxocobalamin prevented mice treated with sodium hydrosulfide from death [104]. Although inhibitors or scavengers may effectively reduce H_2_S concentration levels, they may have undesirable consequences during practical use due to the ubiquity of enzymes and systemic impact, inevitably causing damage to the body. A comprehensive assessment is mandatory to develop a therapeutic agent to eliminate potential side effects. Further translational studies searching for viable therapeutics are necessary.

## 7. Current Status of Evaluating the Sulfur Microbial Diet and Its Association with CRC

Only a limited number of clinical studies have evaluated a dietary pattern associated with microbial sulfur metabolism for the development of CRC, as shown in Table 2. Nguyen, L.H. et al. developed a sulfur microbial dietary scoring system based on dietary elements associated with bacterial species involved in sulfur metabolism. Analyzing serial stool metagenomes and metatranscriptomes from CRC patients in association with sulfur microbial dietary scores, the authors identified that high sulfur microbial dietary scores were associated with increased consumption of high intakes of low-calorie beverages, french fries, red meats, and processed meats and low intakes of fruits, yellow vegetables, whole grains, legumes, leafy vegetables, and cruciferous vegetables [90,105]. Namely, the sulfur microbial diet on long-term adherence was associated with a high concentration of sulfur-metabolizing bacteria in the feces of CRC patients compared to healthy individuals [90]. Furthermore, tight adherence to the microbial sulfur diet was associated with an increased risk of CRC, especially in the distal location [105,106]. Similarly, a large prospective cohort study of women with detailed information on adult and adolescent diets revealed that long-term adherence to a sulfur microbial diet might be associated with an Increased risk of developing adenoma with malignant potentials before age 50 [107]. The authors suggested that the risk might begin as early as adolescence [107].

Yet, the previous studies are based on the hypothesis speculating that a high concentration of the sulfur-metabolizing bacteria may be related to the development of CRC and CRC precursors. Because microbial metabolism in the gut is complex and intertwined with numerous exposomal factors, further clinical studies should be reproduced in different regions and cultures of food habits. Furthermore, one accurate way to determine whether endogenous H_2_S concentration produced by gut microbes causes the carcinogenesis of CRC may be a directly measuring H_2_S concentration in the gut. However, a direct measurement of H_2_S concentration is not available and technically challenging [75]. Thus, it would be essential to develop a diagnostic method to measure H_2_S concentration to assess the relationship between dietary habits and bacterial metabolism.

## 8. Conclusions

It is tragic to face the rise of EOCRC worldwide. There is no doubt the need to address the issue of EOCRC urgently. The priority would be increasing public awareness of the harmful impact of ultra-processed food or Western diet, particularly in children and adolescents. However, a diet as an exposomal element is only speculated. The mechanism of how various dietary elements have positive or negative interactions with gut microbiota is one key element to understanding tumorigenesis occurring early in life. Sulfur metabolism occurring in the gut by microbiota has been suggested as a critical product of the Western diet directly connected to carcinogenesis. Depending on its concentration, the intricate activity of H_2_S provides profound insights into preventive measures with daily dietary management for tumor-targeting therapeutics. Further research is necessary to understand the role of sulfur-metabolizing bacteria in the association between diet and CRC development.

## Figures and Tables

**Table 1:** The difference in sporadic tumors between EOCRC and LOCRC.

	EOCRC	LOCRC
Tumor location	Left colon and rectum	Right colon
Prognosis	Poor prognosis with high metastatic disease at diagnosis	Low frequency of synchronous or metachronous tumors
Molecular aberration	- More likely to be microsatellite stable - Low frequency of CpG island methylator phenotype - Low BRAF mutation - Absent MLH1 expression - Presence of MSH2 inactivation - Long interspersed nuclear elements (LINE-1) hypomethylation	- High frequency of CpG island methylator phenotype - High BRAF mutation - MLH 1 hypermethylation present

Table 1 is created based on the summary of the published articles by Stoffel and Murphy [14], Ballester, V. et al. [28], and Zaborowski et al. [36].

**Table 2:** Clinical studies are evaluating the association between the sulfur microbial diet and CRC.

Authors	Year	Study Type	Cohort	Comparatives	Findings
Magee, E.A. et al. [73]	2000	Clinical trial	5 healthy men	The intervention of change in dietary components: vegetarian diet vs. high meat diet - Measurement of fecal sulfide concentrations for each type of diet	- High concentration of sulfide correlated with protein digestion
Sikavi, D.R. et al. [91]	2021	Prospective observational	51,529 men enrolled in the Health Professionals Follow-up Study	Cancer tissues obtained from CRC patients - Intratumoral variations of microbial species in the CRC subtypes - Intratumoral *Bifidobacterium* spp. (+) tumors vs. (−) tumors	- Sulfur microbial dietary pattern associated with an increased abundance of cancer-associated sulfur-metabolizing bacteria may be more strongly associated with prostaglandin synthase 2 high tumors - High sulfur microbial diet socres associated with distal CRC in the absence of intratumoral *Bifidobacterium* spp.
Nguyen, L.H. et al. [76]	2020	Prospective observational	51,529 men enrolled in the Health Professionals Follow-up Study	CRC patients vs. Healthy individuals - Sulfur microbial dietary score - Serial stool metagenomes and metatranscriptomes	- High sulfur microbial diet scores are associated with increased consumption of processed meats and low-calorie drinks and low consumption of vegetables and legumes - Increased sulfur microbial diet scores were associated with a risk of distal colon and rectal cancers (RR 1.43, 95% CI 1.14–1.81, *p*-trend = 0.002)
Wang, Y. et al. [90]	2021	Prospective observational	- 51,529 male from Health Professionals Follow-up Study - 121,700 females from Nurses’ Health Study - 116,429 females from Nurses’ Health Study II	CRC patients vs. Healthy individuals - Sulfur microbial dietary score - Serial stool metagenomes and metatranscriptomes	- High intakes of low-calorie beverages, french fries, red meats, and processed meats and low intakes of fruits, yellow vegetables, whole grains, legumes, leafy vegetables, and cruciferous vegetables characterize high sulfur microbial diet scores - Greater adherence to the sulfur microbial diet associated with increased risk of CRC (HR 1.27, 95% CI 1.12–1.44, *p* < 0.001) - Increased risk of CRC in distal location (HR 1.25, 95% CI 1.05–1.50, *p* = 0.02)
Nguyen, L.H. et al. [92]	2021	Prospective observational	- 116,429 female aged 25 to 42 years	Individuals with polyps vs without polyps	- Long-term adherence to a sulfur microbial diet may be associated with increased risk for adenoma before age 50

## Data Availability

No new data were created or analyzed in this study. Data sharing is not applicable to this article.
